# Development of an immunogenicity score for HLA‐DQ eplets: A conceptual study

**DOI:** 10.1111/tan.14110

**Published:** 2020-10-28

**Authors:** Lara Schawalder, Gideon Hönger, Marc Kleiser, Michelle R. van Heck, Loes A. L. van de Pasch, Sanne Vendelbosch, Erik H. Rozemuller, Stefan Schaub

**Affiliations:** ^1^ Clinic for Transplantation Immunology and Nephrology, University Hospital Basel Basel Switzerland; ^2^ Transplantation Immunology, Department of Biomedicine University of Basel Basel Switzerland; ^3^ HLA‐Diagnostics and Immungenetics, Department of Laboratory Medicine University Hospital Basel Basel Switzerland; ^4^ GenDX Utrecht The Netherlands

**Keywords:** eplets, HLA‐DQ, immunogenicity

## Abstract

Eplets are defined as distinct amino acid configurations on the surface of HLA molecules. The aim of this study was to estimate the immunogenicity of HLA‐DQ eplets in a cohort of 221 pregnancies with HLA‐DQ mismatches. We defined the immunogenicity of an eplet by the frequency of antibody responses against it. Around 90% of all listed DQB1 or DQA1 eplets were at least five times mismatched and thus included for the calculation of their immunogenicity. The DQB1 eplets with the five highest immunogenicity scores were 55PP, 52PR, 52PQ, 85VG and 45EV; 25% of all DQB1 eplets were not reacting. The DQA1 eplets with the five highest immunogenicity scores were 25YS, 47QL, 55RR, 187T and 18S; 17% of all DQA1 eplets were not reacting. The immunogenicity score had a slightly higher area under the curve to predict development of child‐specific antibodies than various molecular mismatch scores (eg, eplet mismatch load, amino acid mismatch load). Overlapping eplets were identified as a barrier to unambiguously assign the immunogenicity score based on HLA antibody reaction patterns. In this conceptual study, we explored the immunogenicity of HLA‐DQ eplets and created a map of potentially immunogenic regions on HLA‐DQ molecules, which requires validation in clinical transplant cohorts.

**Abbreviations**
AUC: Area under the curve
CSA: child‐specific antibodies
DSA: donor‐specific HLA antibodies
MFI: mean fluorescent intensity
SAB: single antigen beads

## 1 INTRODUCTION

Disparities between HLA molecules of the donor and recipient are the major driving force for rejection in solid organ transplantation.^1^ Traditionally, entire HLA molecules between the donor and recipients were compared and the number of mismatches counted. This way to assess the compatibility is still an essential part for organ allocation and selection of living donors. However, already decades ago many studies clearly highlighted that not every HLA mismatch is equivalent and that rather distinct molecular structures on the surface of individual HLA molecules are responsible for the induction of an immune response.^2^, ^3^

Duquesnoy et al developed a concept defining amino acid configurations on the surface of HLA molecules as unique parts (ie, eplets).^4^, ^5^, ^6^ Rather than counting entire HLA molecule differences, this approach dissects HLA molecules into eplets for subsequent comparison. Several studies showed that the load of HLA‐DR/DQ eplet mismatches is much better to predict the development of de novo donor‐specific antibodies (DSA) against HLA‐DR/DQ after solid organ transplantation than conventional HLA mismatch counts.^7^, ^8^, ^9^, ^10^ Another approach to define HLA disparities on a molecular level counts single amino acid mismatches and/or their physicochemical properties.^11^, ^12^, ^13^ Both molecular mismatch assessments (ie, eplet mismatch load, amino acid mismatch load) were found to correlate with each other and have similar potential to predict the development of de novo DR‐/DQ‐DSA.^14^

Although molecular mismatch loads are more precise to predict an immune response than conventional HLA mismatch counts, the next logical step to further improve this concept requires the assignment of an “immunogenicity tag” to each individual eplet or amino acid configuration.^15^, ^16^ Unfortunately, this task is complex and still in its infancy. The initial approach defined the immunogenicity of eplets by the frequency of antibody responses against them in transplant recipients (reviewed in Reference 17). The key problem of this approach are confounders such as different immunosuppression and the complexity in case of many HLA mismatches. Very recently, Tambur et al used preferably homozygous transplant recipients developing de novo DQ‐DSA against only one of two mismatched HLA‐DQ molecules.^18^ This very elegant in vivo experimental setup has the advantage of a built‐in control for major confounders (eg, immunosuppression, characteristics of the immune system), but likely requires an international multicenter effort to compile sufficient informative cases.

The human pregnancy has been instrumental to investigate humoral immune responses against HLA molecules.^1^, ^19^, ^20^, ^21^, ^22^ It may also serve as an attractive model to define the immunogenicity of individual eplets, because only one HLA molecule mismatch per locus is present and interference by immunosuppressive drugs does not exist. Therefore, we aimed to develop an immunogenicity score for HLA‐DQ eplets and to create a map of immunogenic regions on HLA‐DQ molecules using a pregnancy cohort.

## 2 METHODS

### 2.1 Population and analytical overview

This study was approved by the local ethics committee. After obtaining written informed consent, 301 women giving birth at the University Hospital Basel between September 2009 and April 2011 were enrolled in the study. All women had either their first full‐term pregnancy or had previous children only from the same partner as the current live birth. A blood sample was drawn from the mother between day 1 and 4 after delivery for HLA typing and HLA antibody analysis. Cord blood of the child was obtained immediately after delivery for HLA‐typing.

**FIGURE 1:**
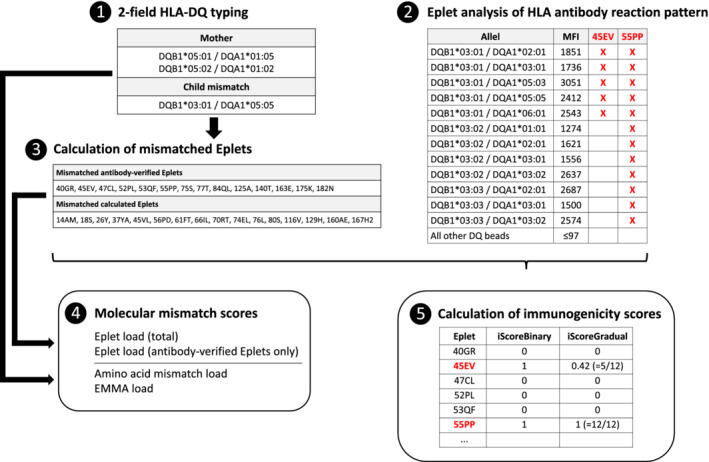
Overview of the analysis and calculation of different scores

The final population for the study included 278 mother‐child pairs with complete high‐resolution DQB1‐ and DQA1‐HLA typing and HLA antibody analysis by single antigen beads (SAB). With these data, we calculated various molecular mismatch scores and two immunogenicity scores. Figure 1 provides an overview of the analyses, which are further detailed in the following paragraphs.

### 2.2 High‐resolution HLA typing

High‐resolution HLA typing of mothers and children was performed by next‐generation sequencing using NGSgo workflow from GenDx (www.gendx.com; Utrecht, The Netherlands) on a MiSeq instrument from Illumina (www.illumina.com; San Diego, California) using MiSeq V2 reagents. The allele calling was done by NGSengine from GenDx using the IMGT/HLA database version 3.33.

### 2.3 HLA antibody analysis and assignment as child specific

HLA antibody analysis was performed by SAB class II (LABScreen SA II Lot 8; OneLambda ThermoFisher, Canoga Park, California) according to the instructions of the manufacturer. Lot 8 consisted of 29 different DQB1/DQA1 heterodimers, the current lot 13 has 28 different DQB1/DQA1 heterodimers. The heterodimer composition of lot 8 and lot 13 is identical, with the exception of one additional heterodimer in lot 8 (DQB1*03:02/DQA1*01:01). SAB with a baseline normalized mean fluorescence intensity (MFI) >500 were considered as positive. Positive reactions against the paternal DQB1 and/or DQA1 alleles were classified as child‐specific antibodies (CSA) and all paternal DQB1 and/or DQA1 carrying SAB had to be positive. Other cutoff definitions had no significant impact on the assignment of CSA (eg, MFI > 300 or ratio above the mother's own class II SAB reactivity).

### 2.4 Molecular mismatch scores

Eplet mismatches (both antibody‐verified and theoretical) were determined according to eplet definitions in HLAMatchmaker version 2.1, which is the most frequently used version in clinical studies.^7^, ^8^, ^9^, ^10^, ^23^ Amino acid mismatches of the paternal DQ heterodimer were determined using amino acid sequences of the IMGT/HLA database version 3.37. A recently published software program called HLA epitope mismatch algorithm (HLA‐EMMA) determines polymorphic solvent accessible amino acid mismatches.^13^ We used the batch analysis feature in software version 1.0 to calculate the number of solvent accessible amino acid mismatches.

### 2.5 Eplet analysis of HLA antibody pattern and calculation of immunogenicity scores

To assess eplet specificities of the identified CSA we analyzed the reactivity pattern of each sample using eplet definitions in HLAMatchmaker version 2.1. This approach first separates SAB into two groups: (a) not reacting SAB (MFI < 500) and (b) reacting SAB (MFI ≥ 500). All eplets expressed on not reacting SAB are eliminated from the pool of potentially targeted eplets. The remaining eplets are represented on at least one SAB and are analyzed regarding their antibody reactivity. This approach assumes that a targeted eplet can only be assigned when all SAB carrying it are reacting.

To assign the relative immunogenicity of the identified eplets, we used two different scores (Figure 1). For calculation of the “binary immunogenicity score” (iScoreBinary), the frequency of an eplet‐specific antibody response was used. For example, eplet 45EV was mismatched in 33 pregnancies and presumably 45EV‐specific CSA were detected in eight mothers, giving an iScoreBinary score of 8/33 or 0.242.

In most cases, several eplets could potentially explain the HLA antibody reactivity pattern (Figure 1). For calculation of the iScoreBinary, every potentially targeted eplet is regarded as equivalent, whether it is expressed on just one or all reacting SAB. To better account for the likelihood of a truly targeted eplet, we also calculated the “gradual immunogenicity score” (iScoreGradual). The iScoreGradual is calculated as the number of reacting SAB expressing the potentially targeted eplet divided by the total number of reacting SAB. For example, eplet 45EV is expressed on 5/12 reacting SAB giving an iScoreGradual of 0.42 (Figure 1). The total iScoreGradual for each eplet is calculated as the sum of the individual iScoreGradual in all reacting samples. For example, if the 45EV eplet was potentially reacting in six samples with individual iScoreGradual of 0.2, 0.2, 0.42, 0.42, 0.65 and 0.85, the total iScoreGradual is 2.74. In all analyses, the iScoreGruadual represents the total score. Overall, the iScoreBinary might overestimate the immunogenicity of very distinct private eplets accompanying broader eplets, while the iScoreGradual might underestimate their immunogenicity.

### 2.6 Visualization of immunogenic regions on the HLA‐DQ molecule

Many eplets are composed of overlapping amino acid positions, which makes it very difficult to visualize the immunogenicity of such eplets. Therefore, we had to split the eplets into the corresponding individual amino acid positions giving each one the iScoreGradual of the underlying eplet. All individual iScoreGradual of a specific position and a specific amino acid were then summed up. For example, the overlapping eplets 55PP and 55RL consist of two amino acids (55P and 56P, as well as 55R and 56L, respectively). The total score for position 55 was therefore calculated as the sum of 55P and 55R inferred by the iScoreGradual of 55PP and 55RL. Finally, each amino acid position and each specific amino acid at this position obtained a score. Overall, 39 individual positions were found on DQB1, 32/39 had different amino acids; 27 individual positions were found on DQA1, 22/27 had different amino acids. We arbitrarily defined three groups based on the quartiles of the position score: (a) high immunogenicity (top quartile), (b) intermediate immunogenicity (two middle quartiles), (c) low immunogenicity (lowest quartile). We used PyMOL software (Schrödinger Inc.) to visualize the immunogenicity of individual positions and amino acids on a surrogate DQ molecule with available 3D‐structure information (DQB1*06:02/DQA1*01:39 [1UVQ]).

### 2.7 Statistical analysis

We used JMP software (SAS Institute Inc., Cary, North Carolina) for statistical analysis. For categorical data, Fisher's exact test or Pearson's chi‐square test was used. For nonparametric continuous data, the Wilcoxon rank‐sum test was used for analysis and data are presented as median (interquartile range) unless stated otherwise. A *P*‐value <.05 was considered to indicate statistical significance.

## 3 RESULTS

### 3.1 Characteristics of the cohort

The study cohort consists of 278 healthy full‐term pregnancies in a predominantly Caucasian population. In 57 of 278 mother‐child pairs (21%), we found no DQB1/DQA1 mismatches, leaving 221 cases for the final analysis. The median age was 31 years (28‐34). In 142 of 221 cases (64%) it was a first full‐term pregnancy, 79 of 221 mother's (36%) had at least one prior pregnancy from the same father as the current pregnancy. Only 3 of 221 women had additional sensitizing events (ie, blood transfusions).

### 3.2 Frequency and characteristics of DQ heterodimer mismatches

The shared, maternal and paternal HLA haplotypes were segregated. Among the 221 cases, 41 different DQ heterodimer mismatches were noticed. DQ heterodimer with a mismatch frequency > 1% are summarized in Table 1. The three most frequent DQ heterodimer mismatches were DQB1*03:01/DQA1*05:05, DQB1*02:02/DQA1*02:01 and DQB1*06:02/DQA1*01:02. All DQ heterodimer mismatches showed very large differences regarding total eplet and amino acid mismatch loads (Table 1).

**TABLE 1:** Frequency/characteristics of HLA‐DQ heterodimer mismatches and frequency of child‐specific antibodies

DQB1/DQA1 heterodimer	Mismatched, n (%)	Total eplet mismatch load, median (IQR)	Amino acid mismatch load, median (IQR)	CSA +, n (%)	CSA + first pregnancy, n (%)	CSA + ≥second pregnancy, n (%)
DQB1*03:01/DQA1*05:05	27 (12.2%)	13 (12‐20)	18 (13‐39)	8 (30)	2/13 (15)	6/14 (43)
DQB1*02:02/DQA1*02:01	25 (11.3%)	14 (8‐21)	22 (14‐47)	5 (20)	2/19 (11)	3/6 (50)
DQB1*06:02/DQA1*01:02	24 (10.9%)	11 (5‐19)	15 (4‐39)	4 (17)	0/15 (0)	4/9 (44)
DQB1*05:01/DQA1*01:01	19 (8.6%)	20 (9–25)	40 (11‐47)	1 (5)	0/15 (0)	1/4 (25)
DQB1*02:01/DQA1*05:01	17 (7.7%)	13 (8‐19)	21 (13‐39)	2 (12)	2/15 (13)	0/2 (0)
DQB1*03:02/DQA1*03:01	13 (5.9%)	8 (4‐11)	12 (5‐16)	2 (15)	2/7 (29)	0/6 (0)
DQB1*06:03/DQA1*01:03	13 (5.9%)	8 (5‐13)	6 (5–24)	2 (15)	1/8 (13)	1/5 (20)
DQB1*05:02/DQA1*01:02	12 (5.4%)	15 (11‐26)	16 (12‐49)	3 (25)	1/5 (20)	2/7 (29)
DQB1*03:03/DQA1*02:01	7 (3.2%)	7 (4–11)	8 (6‐17)	0	0/4 (0)	0/3 (0)
DQB1*04:02/DQA1*04:01	6 (2.7%)	17 (14‐23)	23 (15‐34)	2 (33)	1/5 (20)	1/1 (100)
DQB1*05:01/DQA1*01:05	6 (2.7%)	14 (3‐18)	16 (3‐24)	0	0/3 (0)	0/3 (0)
DQB1*05:03/DQA1*01:04	6 (2.7%)	11 (8‐28)	14 (10‐51)	0	0/2 (0)	0/4 (0)
DQB1*06:04/DQA1*01:02	4 (1.8%)	5 (4‐13)	5 (4‐28)	1 (25)	1/4 (25)	‐
DQB1*06:09/DQA1*01:02	4 (1.8%)	14 (8–19)	28 (12‐42)	0	0/3 (0)	0/1 (0)
DQB1*03:01/DQA1*03:03	3 (1.4%)	17 (9‐18)	19 (12‐22)	1 (33)	1/2 (50)	0/1 (0)
DQB1*03:03/DQA1*03:02	3 (1.4%)	16 (12‐27)	20 (20‐50)	1 (33)	1/2 (50)	0/1 (0)
DQB1*03:04/DQA1*03:03	3 (1.4%)	11 (8‐17)	20 (12–20)	1 (33)	0/2 (0)	1/1 (100)
DQB1*06:01/DQA1*01:03	3 (1.4%)	10 (2–19)	11 (2‐41)	0	0/1 (0)	0/2 (0)

*Note:* In total, 41 different mismatched DQ heterodimers were noticed in 221 mismatched mother‐child pairs. Eighteen mismatched HLA‐DQ heterodimers with a frequency of >1% are shown. They account for 195/221 HLA‐DQ heterodimer mismatches (88%) and 33/37 cases with child‐specific antibodies (CSA). The remaining four cases with CSA were against the following HLA‐DQ heterodimers: DQB1*03:02/DQA1*03:03 (twice mismatched, once with CSA; both cases are first pregnancies), DQB1*03:04/DQA1*03:01 (once mismatched with CSA; ≥second pregnancy), DQB1*06:03/DQA1*03:03 (once mismatched with CSA; ≥second pregnancy), DQB1*06:04/DQA1*04:04 (once mismatched with CSA; ≥second pregnancy).

### 3.3 Frequency of child‐specific antibodies

Thirty‐seven of 221 cases had CSA (17%). In 29 cases, CSA were directed against DQB1 and DQA1, in 6 cases only against DQB1, and in 2 cases only against DQA1. The frequency of CSA among the 41 different DQ heterodimer mismatches ranged from 0% to 100%. Due to low numbers for many DQ heterodimer mismatches, a statistical evaluation regarding the CSA frequency is not reliable. Table 1 summarized the frequency of CSA among the 18 most prevalent DQ heterodimer mismatches. Overall, the frequency of CSA was significantly higher in ≥second pregnancies compared to first pregnancies (22/79 [28%] vs 15/142 [11%]; *P* = .001). However, on the level of individual DQ heterodimer mismatches, CSA were observed more often in first pregnancies in 5/18 DQ heterodimer mismatches, equally frequent in 5/18 DQ heterodimer mismatches, and less frequent in 8/18 DQ heterodimer mismatches (Table 1).

### 3.4 Molecular mismatch score in mothers with/without child‐specific antibodies

**FIGURE 2:**
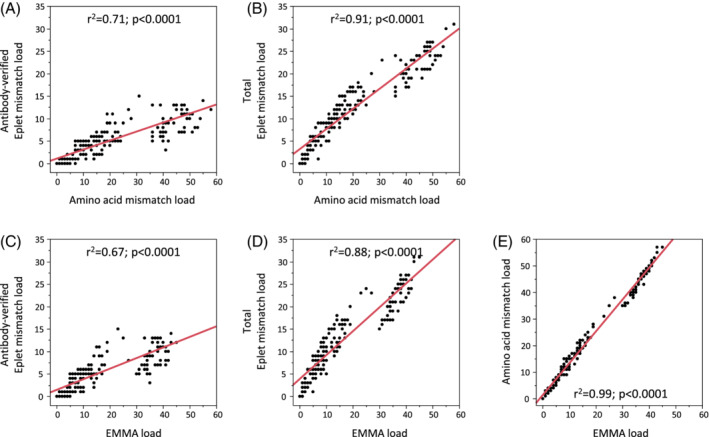
Correlation of different molecular mismatch scores

All three different DQ eplet loads (antibody‐verified, theoretical and total) were significantly higher in mothers, who developed CSA, compare to mothers without CSA. The amino acid mismatch load and the EMMA load for the DQ heterodimer were also significantly higher in CSA positive mothers (Table 2). Despite the significant differences, we noticed large overlaps of all molecular mismatch scores between CSA positive and CSA negative mothers. Furthermore, all molecular mismatch scores correlated strongly with each other (*r* ^2^ between 0.67 and 0.99) (Figure 2).

**TABLE 2:** Molecular mismatch scores in mothers with/without child‐specific antibodies

Molecular mismatch score	CSA + (n = 37)	CSA – (n = 184)	*P*‐level
Eplet load			
Antibody‐verified eplets	6 (4–9)	5 (1‐8)	0.03
Theoretical eplets	8 (6‐12)	6 (4–11)	0.01
Total eplets	15 (10‐23)	11 (6‐19)	0.01
Amino acid mismatch load			
DQB1	13 (8–19)	9 (2‐17)	0.07
DQA1	13 (1‐21)	8 (2–19)	0.18
DQB1/DQA1 heterodimer	20 (13‐42)	15 (7‐39)	0.02
EMMA load			
DQB1	9 (6–17)	7 (1‐15)	0.05
DQA1	10 (1‐17)	6 (1–15)	0.18
DQB1/DQA1 heterodimer	14 (9‐35)	10 (5–33)	0.02

Abbreviations: CSA, child‐specific antibodies; EMMA, epitope mismatch algorithm.

### 3.5 Correlation of DQ eplet frequency in the population and on the SAB panel

**FIGURE 3:**
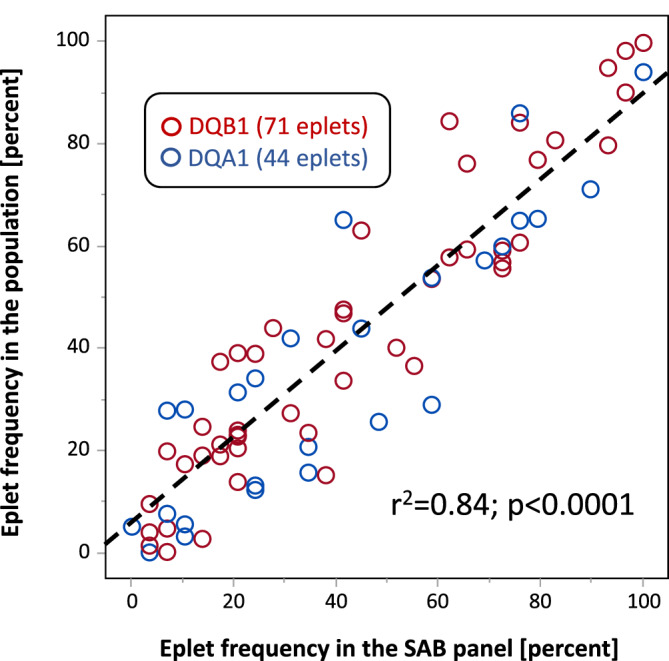
Correlation of the HLA‐DQ eplet frequency in the population (n = 834 haplotypes) and on the used SAB panel lot 8 (n = 29 HLA‐DQ beads)

The population of 278 pregnancies consists of 834 distinct haplotypes, which were used to calculate the population frequency of DQB1 and DQA1 alleles. Similarly, the SAB lot 8 consists of 29 HLA‐DQ dimer, which were used to calculate the DQB1 and DQA1 allele frequency on the SAB panel. Based on these data, we determined the DQB1 and DQA1 eplet frequency in the population and on the SAB panel, which showed a strong correlation (*r* ^2^ = 0.84) (Figure 3).

### 3.6 Immunogenicity scores of DQB1 and DQA1 eplets

The used eplet definition from HLAMatchmaker V2.1 includes 71 DQB1 eplets (30 antibody‐verified plus 41 theoretical). In our cohort, 63/71 DQB1 eplets (89%) were at least five times mismatched and thus included for the calculation of the two immunogenicity scores (ie, iScoreBinary and iScoreGradual). These 63 eplets included all antibody‐verified DQB1 eplets. Forty‐five of 63 DQB1 eplets (71%) were reacting, while 18/63 DQB1 eplets (29%) were not reacting (Table 3). The DQB1 eplets with the five highest iScoreGradual were 55PP, 52PR, 52PQ, 85VG and 45EV. Antibody‐verified eplets accounted for 16/20 eplets (80%) with the highest iScoreGradual.

**TABLE 3:** DQB1 eplet immunogenicity

Reacting eplets					Not reacting eplets				
Eplet	Eplet type	N mismatched	iScore binary	iScore gradual	Eplet	Eplet type	N mismatched	iScore binary	iScore gradual
55PP	AbV	32	0.219	5.476	66D	T	39	0	0
52PR	AbV	36	0.139	4.489	75V	T	37	0	0
52PQ	AbV	36	0.167	3.993	26L	T	35	0	0
85VG	AbV	36	0.167	3.993	66DR	T	35	0	0
45EV	AbV	33	0.242	3.394	13GM	T	32	0	0
125GQ	T	39	0.154	3.018	14AM	T	31	0	0
84QL	AbV	27	0.111	3	26Y	T	31	0	0
125A	AbV	27	0.111	3	167H2	T	31	0	0
85VA	AbV	37	0.162	2.995	140A	AbV	23	0	0
45GV	AbV	22	0.136	2.496	182S	AbV	23	0	0
67VG	T	39	0.154	2.496	167R	T	11	0	0
45GE	AbV	34	0.147	2.49	66EV	T	10	0	0
55LPA	AbV	34	0.147	2.49	116V	T	8	0	0
74AVR	AbV	34	0.147	2.49	9Y	T	6	0	0
87F	AbV	36	0.167	2.012	28T	AbV	6	0	0
52PL	AbV	32	0.063	1.524	46VY	AbV	6	0	0
140T	AbV	32	0.063	1.524	52P	AbV	6	0	0
182N	AbV	32	0.063	1.524	185T	T	6	0	0
70GT	T	34	0.176	1.508					
85VY	T	40	0.075	1.11					
9F	T	31	0.097	1.018					
26G	T	40	0.05	1					
74SV	AbV	40	0.05	1					
77T	AbV	22	0.045	1					
55RL	AbV	7	0.143	1					
66DI	AbV	7	0.143	1					
70ED	T	7	0.143	1					
56PA	T	40	0.05	0.958					
37YA	T	24	0.042	0.955					
14GL	T	36	0.111	0.889					
37YV	T	36	0.111	0.889					
74SR	AbV	36	0.111	0.889					
116I	AbV	36	0.111	0.889					
125S	AbV	36	0.111	0.889					
185I	T	22	0.091	0.865					
70RT	T	30	0.033	0.833					
74EL	T	26	0.038	0.818					
77R	AbV	35	0.029	0.778					
56PV	AbV	29	0.069	0.606					
86G	T	9	0.222	0.582					
130Q	T	9	0.222	0.582					
56PD	T	26	0.038	0.545					
135G	T	27	0.185	0.355					
125SH	T	13	0.231	0.333					
56PS	T	12	0.25	0.333					

*Note:* Sixty‐three DQB1 eplets were at least five times mismatched and included for the calculation of the two immunogenicity scores (ie, iScoreBinary and iScoreGradual). Forty‐five of 63 DQB1 eplets (71%) were reacting, while 18/63 DQB1 eplets (29%) were not reacting. Reacting eplets are sorted by the iScoreGradual.

Abbreviations: AbV, antibody‐verified eplet; T, theoretical eplet.

The used eplet definition from HLAMatchmaker V2.1 includes 44 DQA1 eplets (11 antibody‐verified plus 33 theoretical). In our cohort, 41/44 DQA1 eplets (93%) were at least five times mismatched and thus included for the calculation of the two immunogenicity scores. These 41 eplets included all antibody‐verified DQA1 eplets. Thirty‐four of 41 DQA1 eplets (83%) were reacting, while 7/41 DQA1 eplets (17%) were not reacting (Table 4). The DQA1 eplets with the five highest iScoreGradual were 25YS, 47QL, 55RR, 187T and 18S. Antibody‐verified eplets accounted for 8/20 eplets (40%) with the highest iScoreGradual.

**TABLE 4:** DQA1 eplet immunogenicity

Reacting eplets					Not reacting eplets				
Eplet	Eplet type	N mismatched	iScore binary	iScore gradual	Eplet	Eplet type	N mismatched	iScore binary	iScore gradual
25YS	AbV	25	0.24	3.807	25FT	T	38	0	0
47QL	AbV	25	0.24	3.807	25YT	T	27	0	0
52RR	AbV	25	0.24	3.807	129H	T	19	0	0
187T	T	25	0.24	3.807	160AD	T	16	0	0
18S	T	27	0.111	2.856	2G	T	12	0	0
45VL	T	27	0.111	2.856	199T	T	12	0	0
61FT	T	27	0.111	2.856	187A	T	5	0	0
80S	T	27	0.111	2.856					
66IL	T	29	0.103	2.43					
47KHL	AbV	32	0.125	1.983					
40GR	AbV	38	0.132	1.947					
47CL	AbV	38	0.132	1.947					
53QF	AbV	38	0.132	1.947					
76L	T	45	0.067	1.857					
40ERV	AbV	39	0.077	1.769					
40ER	T	24	0.083	1.653					
175E	T	39	0.051	1.44					
75I	T	14	0.071	0.963					
163I	T	14	0.071	0.963					
160D	T	13	0.308	0.911					
75S	AbV	33	0.121	0.786					
163E	AbV	33	0.121	0.786					
175K	AbV	33	0.121	0.786					
160AE	T	32	0.125	0.523					
75IL	T	39	0.026	0.476					
18F	T	36	0.028	0.474					
52SK	T	36	0.028	0.474					
64RM	T	36	0.028	0.474					
80Y	T	36	0.028	0.474					
41KA	T	16	0.125	0.417					
130A	T	16	0.125	0.417					
41RA	T	37	0.027	0.368					
130S	T	37	0.027	0.368					
66IT	T	10	0.1	0.188					

*Note:* Forty‐one DQA1 eplets were at least five times mismatched and included for the calculation of the two immunogenicity scores (ie, iScoreBinary and iScoreGradual). Thirty‐four of 41 DQA1 eplets (83%) were reacting, while 7/41 DQB1 eplets (17%) were not reacting. Reacting eplets are sorted by the iScoreGradual.

Abbreviations: AbV, antibody‐verified eplet; T, theoretical eplet.

We found a significant, but moderate strong correlation between the iScoreBinary and the iScoreGradual for DQB1 (*r* ^2^ = 0.42; *P* < .0001); the correlation for DQA1 was strong (*r* ^2^ = 0.82; *P* < .0001).

Next, we performed the calculation including only first pregnancy cases (n = 142). There was a significant correlation between the scores calculated from the whole cohort and only from first pregnancies (iScoreGradual: *r* ^2^ = 0.45; *P* < .0001 and iScoreBinary: *r* ^2^ = 0.68; *P* < .0001). On an individual eplet level, 12 of the 20 top‐ranked DQB1 eplets from the whole cohort were also among the 20 top‐ranked DQB1 eplets from the first pregnancy cases. There were 13 additional eplets assigned as not reacting, mostly with low scores in the whole cohort. Regarding DQA1, 14 of the 20 top‐ranked eplets from the whole cohort were also among the 20 top‐ranked eplets from the first pregnancy cases. There were 14 additional eplets assigned as not reacting, mostly with low scores in the whole cohort.

### 3.7 Limitation of the used approach to define the immunogenicity of DQ eplets

**FIGURE 4:**
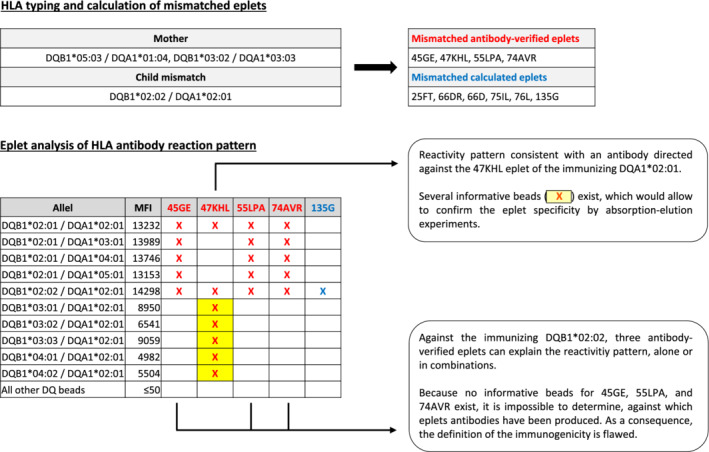
Limitation of the used approach to define the immunogenicity of HLA‐DQ eplets. This illustrative example highlights a key limitation of the used approach

While performing the study, we encountered a major limitation of the approach to define the immunogenicity of eplets via the analysis of the HLA antibody reactivity pattern. If several overlapping eplets can explain the reactivity pattern, then the assignment of the “true” targeted eplet is often impossible and thus the calculation of the immunogenicity is flawed. Absorption‐elution can be used to dissect an HLA antibody mixture and to isolate individual HLA antibodies allowing to characterize their targeting eplet. However, this requires the existence of informative beads, which only carry the eplet of interest. An illustrative example is detailed in Figure 4.

In our study, the 37 cases with CSA had in total 254 mismatched eplets, which were potential targets according to the HLA antibody reactivity pattern (median five eplets per case [IQR 3‐9]). Only 19/254 potentially targeted eplets (7.5%) in 17/37 patients (46%) had informative beads, which would have allowed performing absorption‐elution experiments to define the “true” targeted eplets.

### 3.8 Prediction of child‐specific antibodies by different scores

The diagnostic characteristics of the different molecular mismatch scores and the immunogenicity scores are summarized in Table 5. Overall, the AUC's were low in the range of 0.62 to 0.68. The negative predictive values were high in the range of 89% to 97%, but the positive predictive value was very low between 21% and 27%.

**TABLE 5:** Prediction of child‐specific antibodies by different scores

Parameter	AUC	*P*‐level	Cutoff	Sensitivity (%)	Specificity (%)	NPV (%)	PPV (%)
Antibody‐verified eplet load	0.62	0.04	3	89	34	94	21
Total eplet load	0.63	0.02	8	94	32	97	22
Total amino acid load	0.62	0.06	10	92	33	95	22
EMMA load	0.62	0.07	6	97	28	98	21
iScoreBinary	0.66	0.002	1.45	57	68	89	27
iScoreGradual	0.68	0.002	12.14	81	49	93	24

*Note:* Receiver operating characteristic (ROC) curves were used to define the area under the curve (AUC). Negative predictive values (NPV) and positive predictive values (PPV) were calculated for the best cutoff determined by the highest value of the sum of sensitivity + specificity – 1.

Next, we combined the two parameters with the highest AUC from the molecular mismatch scores (ie, total eplet load) and the immunogenicity scores (ie, iScoreGradual). Thirty of 120 mothers having both parameters above the cutoff (25%) developed CSA compared to only 2/57 mothers (3.5%) having both parameters below the cutoff. The relative risk to develop CSA when both parameters were above the cutoff was 7.13 (95% CI 1.76‐28.78) (*P* = .006).

### 3.9 Visualization of immunogenic regions on DQ molecules

**FIGURE 5:**
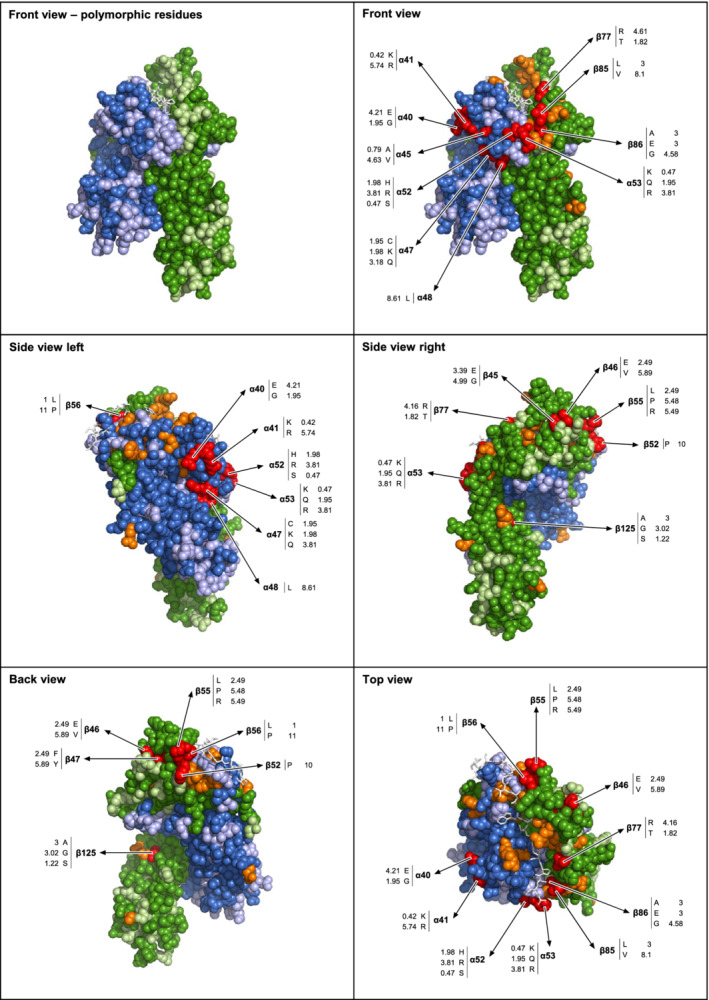
Visualization of immunogenic regions on HLA‐DQ molecules. We used DQB1*06:02/DQA1*01:39 (1UVQ) as a surrogate molecule. DQA1 is colored in blue; polymorphic residues in light blue. DQB1 is colored in green; polymorphic residues in light green. For both DQA1 and DQB1, position of high immunogenicity are marked in red (including the immunogenicity value for individual amino acids), position of intermediate immunogenicity are marked in orange. Polymorphic position of low or absent immunogenicity are not marked and they maintained their light blue or light green color

As detailed in the Methods section, we defined positions of high, intermediate and low immunogenicity. Positions of high immunogenicity are highlighted in red and the immunogenicity value of individual amino acids at those positions are appended (Figure 5).

## 4 DISCUSSION

Defining the immunogenicity of eplets or specific amino acid configuration is an unmet need in solid organ transplantation. In this conceptual study, we tried to develop an immunogenicity score for HLA‐DQ eplets and to create a map of immunogenic regions on HLA‐DQ molecules. Although the immunogenicity score (ie, iScoreGradual) seems to be slightly better than molecular mismatch loads for prediction of CSA, we found a significant limitation of the used approach.

Indeed, it is very challenging to clearly determine the targeted eplet based on an antibody reaction pattern. So far, most investigators either reduced the complexity by focusing only on antibody‐verified eplets or selected the putative target based on the simplest eplet combination, which was regarded as the most likely one.^9^, ^23^, ^24^ Therefore, such approaches have inherent biases. In this study, we aimed to explore the immunogenicity of all HLA‐DQ eplets in an unbiased manner. Both the iScoreBinary and the iScoreGradual have also significant limitations as detailed in the methods section, but we believe that for this study design the iScoreGradual currently offers the most balanced information regarding the eplet immunogenicity.

Despite the rather simple constellation in the pregnancy model having only one mismatched HLA‐DQ heterodimer, the reaction patterns were often complex, and overlapping eplets confounded the calculation of their immunogenicity. Informative beads allowing to isolate HLA antibodies by absorption‐elution were only found for 7.5% of all potentially targeted eplets. The antibody reaction pattern analysis might be even more complicated in a transplant setting with two mismatched haplotypes. Ideally, the antibody‐producing cells are isolated to a single cell level allowing to study the targeted epitope of such monoclonal antibodies in detail. Kramer et al recently described the methodology of this approach for antibodies directed against HLA‐DR molecules.^21^ They explored the molecular targets of five monoclonal antibodies derived from memory B‐cells of three individuals immunized by pregnancies. While 2/5 molecular targets corresponded to known eplets, the remaining ones could not be assigned to an eplet defined in the epitope registry (www.epregistry.com.br).

It is clear that the compilation of a detailed and accurate list of eplets as well as their calling by software programs are key components for all molecular mismatch scores and the assignment of the immunogenicity of individual eplets.^25^ As shown by Kramer et al as well as Tambur et al, the current eplet lists need further refinements and verification.^18^, ^21^ Our data may also help in this regard. If confirmed in other studies, not reacting eplets might be removed from the eplet lists. Furthermore, eplets with a putative high immunogenicity might be candidates for further in‐detail investigations to determine or validate their precise amino acid configurations.

McCaughan et al described two HLA‐DQ heterodimer containing risk epitope mismatches (ie, DQA1*05 combined with DQB1*03:01 [=DQ7] or DQ2), which are associated with a higher risk of de novo DQ‐DSA development in heart and lung transplant recipients.^23^ Interestingly, these HLA‐DQ molecules contain several top‐ranked eplets of our study (eg, DQB1*03:01 contains 55PP [rank #1], 45EV [rank #5], 84QL [rank #7] and 125A [rank #8]) (Table 3). Furthermore, Snanoudj et al found a higher risk for development of de novo DSA against HLA‐DQB1*03:01, if eplet mismatches against 55PP, 70RT or 74EL were present.^9^ Overall, it seems that the 55PP eplet is a major determinant for induction of a humoral immune response against HLA‐DQB1*03:01.

We found a strong correlation among the different molecular mismatch scores and they had very similar diagnostic characteristics to predict the development of CSA, which is in line with a recent report investigating de novo DR‐/DQ‐DSA development in 596 renal transplant recipients.^14^ In another study by Wiebe et al, a single DQ‐molecule eplet load of 9 was found as the best cutoff to predict development of de novo DQ‐DSA in real allograft recipients.^7^ In our pregnancy cohort, the best eplet load cutoff for prediction of CSA was 8. This suggests that the molecular mismatch scores are quite robust and valid in both the transplant and pregnancy context.

The AUC for all molecular mismatch scores and the immunogenicity scores were only moderate in the range of 0.62 to 0.68. All scores had very high negative predictive values, but very low positive predictive values. In other words, a low score indicates a very low risk for development of CSA, almost close to the level of no HLA mismatch. By contrast, a score above the cutoff represents a significantly higher risk, but there is still a large proportion of women having a high score without developing CSA. A sim2ilar observation was made in a transplant cohort, in which recipients with a low total HLA‐DR/DQ eplet load (ie, <7 for HLA‐DR, <9 for HLA‐DQ) had a very low incidence of de novo DR‐/DQ‐DSA similar to recipients without HLA‐DR/DQ mismatches.^7^ It seems, that the currently best use of these scores is to define low risk mismatch constellation.

We were hoping that the immunogenicity scores perform much better than the molecular mismatch loads for prediction of CSA. Unfortunately, this was not true and the AUC were only marginally higher. We believe that this is mostly related to the difficulty to properly assign the targeted eplets as discussed above, which flaws the calculation of the immunogenicity scores. Nevertheless, it would be interesting to investigate the performance of the immunogenicity scores in transplant cohorts, where molecular mismatch scores for HLA‐DQ already reached AUC between 0.72 and 0.84.^7^

To the best of our knowledge, this is the first study aiming to investigate the immunogenicity of the full HLA‐DQ eplet set of HLAMatchmaker version 2.1 and to create a map of immunogenic regions on HLA‐DQ molecules in a systematic manner. The study has certain limitations. First, the results need to be validated in independent cohorts, preferably transplant recipients, because immunization due to pregnancy and solid organ transplantation might be different. Second, inclusion of women with ≥second pregnancies might introduce a bias toward repeatedly exposed eplets. Third, despite investigating 221 cases with HLA‐DQ mismatches, the immunogenicity of eplets with a low frequency could not be assessed. Fourth, as the cohort consists of almost exclusively Caucasian couples, eplets more common in other ethnicities are not well represented. Fifth, the HLA‐DQ heterodimer composition in the SAB panel (eg, from different vendors) influences the iScoreGradual giving more weight to eplets present on several SAB. However, the eplet frequency in the population strongly correlated with the eplet frequency on the used HLA‐DQ SAB panel, suggesting that the number of reacting SAB is a reasonable surrogate of the eplet representation in the population. Finally, we used eplet definitions of HLAMatchmaker version 2.1 and the results might be different with other versions.

In conclusion, in this conceptual study, we explored the immunogenicity of HLA‐DQ eplets and created a map of potentially immunogenic regions on HLA‐DQ molecules, which requires validation in clinical transplant cohorts.

## CONFLICT OF INTEREST

The authors have declared no conflicting interests.

## AUTHORS CONTRIBUTIONS

L.S., G.H., S.S. participated in research design; all authors participated in writing of the paper; all authors participated in the performance of the research; L.S., G.H., S.S. participated in data analysis; All authors read and approved the final manuscript.

## Supporting information


**Table S1** DQB1 eplet immunogenicity. Sixty‐three DQB1 eplets were at least five times mismatched and included for the calculation of the two immunogenicity scores (ie, iScoreBinary and iScoreGradual). Forty‐five of 63 DQB1 eplets (71%) were reacting, while 18 of 63 DQB1 eplets (29%) were not reacting. Reacting eplets are sorted by the iScoreBinary. AbV, antibody‐verified eplet; T, theoretical eplet
**Table S2**. DQA1 Eplet immunogenicity. Forty‐one DQA1 eplets were at least five times mismatched and included for the calculation of the two immunogenicity scores (ie, iScoreBinary and iScoreGradual). Thirty‐four of 41 DQA1 eplets (83%) were reacting, while 7 of 41 DQB1 eplets (17%) were not reacting. Reacting eplets are sorted by the iScoreBinary. AbV, antibody‐verified eplet; T, theoretical eplet

## Data Availability

The data that support the findings of this study are available from the corresponding author upon reasonable request.
