# Supplementary material for: Development of an immunogenicity score for HLA‐DQ eplets: A conceptual study
Source: HLA. 2020 Oct 28;97(1):30–43. doi: 10.1111/tan.14110 (PMC7756751; doi:10.1111/tan.14110)
Supplement: Supplementary file 1 — Table S1 DQB1 eplet immunogenicity. Sixty‐three DQB1 eplets were at least five times mismatched and included for the calculation of the two immunogenicity scores (ie, iScoreBinary and iScoreGradual). Forty‐five of 63 DQB1 eplets (71%) were reacting, while 18 of 63 DQB1 eplets (29%) were not reacting. Reacting eplets are sorted by the iScoreBinary. AbV, antibody‐verified eplet; T, theoretical eplet Table S2. DQA1 Eplet immunogenicity. Forty‐one DQA1 eplets were at least five times mismatched and included for the calculation of the two immunogenicity scores (ie, iScoreBinary and iScoreGradual). Thirty‐four of 41 DQA1 eplets (83%) were reacting, while 7 of 41 DQB1 eplets (17%) were not reacting. Reacting eplets are sorted by the iScoreBinary. AbV, antibody‐verified eplet; T, theoretical eplet [file TAN-97-30-s001.pdf]

# Supplementary Tables

For Review Only

**Supplementary Table 1. DQB1 eplet immunogenicity**. Sixty-three DQB1 eplets were at least 5 times mismatched and included for the calculation of the two immunogenicity scores (i.e. iScoreBinary and iScoreGradual). Forty-five of 63 DQB1 eplets (71%) were reacting, while 18/63 DQB1 eplets (29%) were not reacting. Reacting eplets are sorted by the iScoreBinary. AbV=antibody-verified eplet, T=theoretical eplet.

| Reacting eplets | | | | |
|---|---|---|---|---|
| Eplet | Eplet type | N mismatched | iScore Binary | iScore Gradual |
| 56PS | T | 12 | 0.25 | 0.333 |
| 45EV | AbV | 33 | 0.242 | 3.394 |
| 125SH | T | 13 | 0.231 | 0.333 |
| 86G | T | 9 | 0.222 | 0.582 |
| 130Q | T | 9 | 0.222 | 0.582 |
| 55PP | AbV | 32 | 0.219 | 5.476 |
| 135G | T | 27 | 0.185 | 0.355 |
| 70GT | T | 34 | 0.176 | 1.508 |
| 52PQ | AbV | 36 | 0.167 | 3.993 |
| 85VG | AbV | 36 | 0.167 | 3.993 |
| 87F | AbV | 36 | 0.167 | 2.012 |
| 85VA | AbV | 37 | 0.162 | 2.995 |
| 125GQ | T | 39 | 0.154 | 3.018 |
| 67VG | T | 39 | 0.154 | 2.496 |
| 45GE | AbV | 34 | 0.147 | 2.49 |
| 55LPA | AbV | 34 | 0.147 | 2.49 |
| 74AVR | AbV | 34 | 0.147 | 2.49 |
| 55RL | AbV | 7 | 0.143 | 1 |
| 66DI | AbV | 7 | 0.143 | 1 |
| 70ED | T | 7 | 0.143 | 1 |
| 52PR | AbV | 36 | 0.139 | 4.489 |
| 45GV | AbV | 22 | 0.136 | 2.496 |
| 84QL | AbV | 27 | 0.111 | 3 |
| 125A | AbV | 27 | 0.111 | 3 |
| 14GL | T | 36 | 0.111 | 0.889 |
| 37YV | T | 36 | 0.111 | 0.889 |
| 74SR | AbV | 36 | 0.111 | 0.889 |
| 116I | AbV | 36 | 0.111 | 0.889 |
| 125S | AbV | 36 | 0.111 | 0.889 |
| 9F | T | 31 | 0.097 | 1.018 |
| 185I | T | 22 | 0.091 | 0.865 |
| 85VY | T | 40 | 0.075 | 1.11 |
| 56PV | AbV | 29 | 0.069 | 0.606 |
| 52PL | AbV | 32 | 0.063 | 1.524 |
| 140T | AbV | 32 | 0.063 | 1.524 |
| 182N | AbV | 32 | 0.063 | 1.524 |
| 26G | T | 40 | 0.05 | 1 |
| 74SV | AbV | 40 | 0.05 | 1 |
| 56PA | T | 40 | 0.05 | 0.958 |
| 77T | AbV | 22 | 0.045 | 1 |
| 37YA | T | 24 | 0.042 | 0.955 |
| 74EL | T | 26 | 0.038 | 0.818 |
| 56PD | T | 26 | 0.038 | 0.545 |
| 70RT | T | 30 | 0.033 | 0.833 |
| 77R | AbV | 35 | 0.029 | 0.778 |

For Review Only

**Supplementary Table 2. DQA1 Eplet immunogenicity**. Forty-one DQA1 eplets were at least 5 times mismatched and included for the calculation of the two immunogenicity scores (i.e. iScoreBinary and iScoreGradual). Thirty-four of 41 DQA1 eplets (83%) were reacting, while 7/41 DQB1 eplets (17%) were not reacting. Reacting eplets are sorted by the iScoreBinary. AbV=antibody-verified eplet, T=theoretical eplet.

| Reacting eplets | | | | |
|---|---|---|---|---|
| **Eplet** | **Eplet type** | **N mismatched** | **iScore Binary** | **iScore Gradual** |
| 160D | T | 13 | 0.308 | 0.911 |
| 25YS | AbV | 25 | 0.24 | 3.807 |
| 47QL | AbV | 25 | 0.24 | 3.807 |
| 52RR | AbV | 25 | 0.24 | 3.807 |
| 187T | T | 25 | 0.24 | 3.807 |
| 40GR | AbV | 38 | 0.132 | 1.947 |
| 47CL | AbV | 38 | 0.132 | 1.947 |
| 53QF | AbV | 38 | 0.132 | 1.947 |
| 47KHL | AbV | 32 | 0.125 | 1.983 |
| 160AE | T | 32 | 0.125 | 0.523 |
| 41KA | T | 16 | 0.125 | 0.417 |
| 130A | T | 16 | 0.125 | 0.417 |
| 75S | AbV | 33 | 0.121 | 0.786 |
| 163E | AbV | 33 | 0.121 | 0.786 |
| 175K | AbV | 33 | 0.121 | 0.786 |
| 18S | T | 27 | 0.111 | 2.856 |
| 45VL | T | 27 | 0.111 | 2.856 |
| 61FT | T | 27 | 0.111 | 2.856 |
| 80S | T | 27 | 0.111 | 2.856 |
| 66IL | T | 29 | 0.103 | 2.43 |
| 66IT | T | 10 | 0.1 | 0.188 |
| 40ER | T | 24 | 0.083 | 1.653 |
| 40ERV | AbV | 39 | 0.077 | 1.769 |
| 75I | T | 14 | 0.071 | 0.963 |
| 163I | T | 14 | 0.071 | 0.963 |
| 76L | T | 45 | 0.067 | 1.857 |
| 175E | T | 39 | 0.051 | 1.44 |
| 18F | T | 36 | 0.028 | 0.474 |
| 52SK | T | 36 | 0.028 | 0.474 |
| 64RM | T | 36 | 0.028 | 0.474 |
| 80Y | T | 36 | 0.028 | 0.474 |
| 41RA | T | 37 | 0.027 | 0.368 |
| 130S | T | 37 | 0.027 | 0.368 |
| 75IL | T | 39 | 0.026 | 0.476 |

For Review Only
